# Real-world efficacy and toxicity of tebentafusp in metastatic uveal melanoma patients treated on the United Kingdom expanded access programme

**DOI:** 10.1093/oncolo/oyag341

**Published:** 2026-08-25

**Authors:** Magdalene Joseph, Jacob Tharakan, Jemma Proudfoot-Jones, Joseph J Sacco, Andrew Furness, David McMahon, Leila Khoja, Lalit Pallan, Patricio Serra, Dulani Ranatunge, Bode Oladipo, Gemma Clements, Lavanya Mariappan, Lindsay Hennah, Toby Talbot, Jenny Nobes, Thomas Carter, Heather Shaw, Paul Nathan

**Affiliations:** Mount Vernon Cancer Centre, Northwood, HA6 2RN, United Kingdom; Mount Vernon Cancer Centre, Northwood, HA6 2RN, United Kingdom; Clatterbridge Cancer Centre, Wirral, CH63 4JY, United Kingdom; Clatterbridge Cancer Centre, Wirral, CH63 4JY, United Kingdom; The Royal Marsden Hospital, London, SW3 6JJ, United Kingdom; The Royal Marsden Hospital, London, SW3 6JJ, United Kingdom; University Hospitals Birmingham NHS Foundation Trust, Birmingham, B15 2GW, United Kingdom; University Hospitals Birmingham NHS Foundation Trust, Birmingham, B15 2GW, United Kingdom; The Christie Hospital, Manchester, M20 4BX, United Kingdom; Castle Hill Hospital, Cottingham, HU16 5JQ, United Kingdom; Belfast City Hospital, Belfast, BT9 7AB, United Kingdom; Belfast City Hospital, Belfast, BT9 7AB, United Kingdom; Newcastle Freeman Hospital, Newcastle upon Tyne, NE7 7DN, United Kingdom; Newcastle Freeman Hospital, Newcastle upon Tyne, NE7 7DN, United Kingdom; Royal Cornwall Hospital, Truro, TR1 3LJ, United Kingdom; Norfolk and Norwich University Hospital, Norwich, NR4 7UY, United Kingdom; Mount Vernon Cancer Centre, Northwood, HA6 2RN, United Kingdom; Mount Vernon Cancer Centre, Northwood, HA6 2RN, United Kingdom; University College London Hospitals, London, NW1 2BU, United Kingdom; Mount Vernon Cancer Centre, Northwood, HA6 2RN, United Kingdom; University College London Hospitals, London, NW1 2BU, United Kingdom

**Keywords:** metastatic uveal melanoma, tebentafusp, real-world, expanded access, toxicity, bi-specific T-cell engager, ImmTAC

## Abstract

**Background:**

Metastatic uveal melanoma (mUM) carries a poor prognosis, with low response rates to checkpoint inhibitors. The phase III first-line IMCgp100-202 trial established tebentafusp, a bi-specific T cell engager, as the first treatment to demonstrate a survival benefit in mUM. Here, we report retrospective real-world efficacy and toxicity outcomes from the tebentafusp United Kingdom Expanded Access Programme (UK EAP).

**Patients and methods:**

115 HLA-A*02:01 positive patients with mUM were treated between July 2021 and June 2023. 10 of 18 UK EAP centres submitted data, and outcomes for 88 patients are reported here. Data was collected on demographics, disease characteristics, survival, and treatment toxicities and analyzed to identify factors impacting on median overall survival (mOS), median progression-free survival (mPFS), occurrence of cytokine release syndrome (CRS) and grade 3/4 rash.

**Results:**

71% of patients were treated in the first-line setting, with 94% having liver metastases. At a median follow-up of 12 months (range 0 to 34), mOS was 21 months (95% CI, 17 to NR), and mPFS was 2.76 months (95% CI, 2.53-3.22). The 12-month overall survival rate was 75% (95% CI, 66.11-85.6). Lower lactate dehydrogenase (LDH) and lower liver metastatic burden showed a trend towards improved overall survival, with baseline LDH ≥2× ULN conferring a very poor prognosis. Lower liver metastatic burden also associated with reduced frequency of CRS. No ≥ grade 3 toxicity was observed beyond dose 5.

**Conclusion:**

Tebentafusp on the UK EAP showed comparable real-world efficacy to the phase III first-line trial data, with manageable toxicities having implications for resource planning.

Implications for practice Data from the UK tebentafusp EAP closely mirrored phase III first-line IMCgp100-202 trial data; with a median overall survival of 21 months, median progression-free survival of 2.76 months and 75% 12-month survival. Lower LDH and lower burden of liver metastases were associated with a trend toward improved survival. Patients with a baseline LDH ≥ 2× ULN had a particularly poor outcome. Data provided in this study on toxicities and the requirement for in-patient support during the first 10 doses of treatment will be helpful for resource planning.

## Introduction

Metastatic uveal melanoma (mUM) carries a poor prognosis, with a median overall survival (mOS) of just over 1 year.^1^^,^^2^ It is characterized by a low tumor mutational burden,^3^ GNAQ/GNA11 driver mutations in ∼90% of UM^4^ and is poorly responsive to checkpoint inhibitor-based immunotherapy.^1^^,^^2^

Tebentafusp is a bi-specific T cell engager binding to a gp100 peptide presented by HLA-A*02:01 on cancer cells and CD3 on T cells, creating an activating synapse between T cells and cancer cells.^5^ Tebentafusp was tested in phase II second-line and phase III first-line mUM trials^6–8^ leading to its approval in 2022 by the EMA, MHRA and the FDA; the first approval of a bi-specific T cell engager in a solid tumor and the first approval of any treatment for mUM.

The first-line IMCgp100-202 open-label, randomized phase III trial of tebentafusp versus investigator’s choice of systemic therapy in untreated mUM^7^^,^^8^ was the first phase III randomized trial to show a survival benefit for any systemic therapy in mUM. 73% of patients in the tebentafusp arm were alive at one year compared to 59% in the control cohort (hazard ratio (HR) for death 0.51; 95% CI, 0.37-0.71; *P* < 0.0001).^7^

A more modest median progression-free survival (mPFS) benefit was observed with 31% of patients on tebentafusp remaining progression-free at 6 months, compared to 19% of control group patients (HR for progression 0.73; 95% CI, 0.58-0.94; *P* .01).^7^ Treatment was well tolerated with a 2% discontinuation rate and diminishing severity of toxicity with successive doses during treatment.^7^ Where cytokine release syndrome (CRS) occurred in the tebentafusp arm, it was rarely severe with predominantly grade 1 (12% of patients) or grade 2 (76% of patients) CRS and only 1% incidence of grade 3 CRS. There were no cases of grade 4/5 CRS or any treatment-related deaths in the study.^7^ Of note, both control and tebentafusp treatment arms had a high proportion of patients of PS0 (67% and 76%, respectively) which is higher than might be expected in a real-world setting.

In December 2024, the National Institute for Clinical Excellence (NICE) in the United Kingdom (UK) approved tebentafusp for HLA‑A*02:01-positive unresectable or mUM in adults, without any restrictions on the line of therapy.

Real-world retrospective studies from France, Germany, and Switzerland of patients treated with tebentafusp in first- and second-line settings demonstrated similar or higher mOS and mPFS figures to the first-line IMCgp100-202 trial data.^9^^,^^10^

More recently, the retrospective, multicenter EURACAN study covering 4 European centers and 175 patients treated with tebentafusp found a mOS of 20 months and median PFS of 4 months, again remarkably similar to survival data in the first-line trial.^11^ This parity with trial data is despite pretreatment with checkpoint inhibitors or chemotherapy in over a third of the real-world cohort across studies.^9–11^

Between July 2021 and June 2023, tebentafusp was made available through an EAP to HLA-A*02:01 positive patients with mUM in 18 treating centers in the UK whilst NICE approval was pending. A total of 115 patients were allocated treatment on this programme. Our retrospective study reports the real-world safety and efficacy outcomes for 88 patients, with data from 9 centers covering the majority of patients treated on the programme. Resource utilization and outcomes for patients with lactate dehydrogenase (LDH) ≥ 2× the upper limit of normal (ULN) have not been reported in previous real-world studies.

## Patients and methods

### Study population

Anonymized data for patients treated on the UK tebentafusp EAP was requested from all participating sites, with responses received from 10 of 18 participating sites. The 10 centers contributing data toward this study collectively treated 91 patients of the 115 patients who received tebentafusp through the EAP. Three patients previously treated on trial with tebentafusp were excluded from further analyses. The final cohort consisted of 88 patients from 9 centers.

Inclusion criteria for the EAP included age ≥18 years, a histological (or cytological) diagnosis of mUM, and the presence of the HLA-A*02:01 allele.^12^ Patients had to be able to provide informed consent and adhere to contraceptive requirements on treatment. Pregnant or breast-feeding patients were excluded from treatment.^12^

Patients were additionally required to have an Eastern Cooperative Oncology Group performance status (ECOG PS) score of 0-1 and adequate bone marrow, cardiac, liver, and kidney function as specified.^12^ Patients were ineligible if they had untreated or active central nervous system metastases, were on immunosuppressive doses of corticosteroids, or had active viral infections.^12^ There were no restrictions on prior systemic or loco-regional therapy, though minimum time intervals from the last line of therapy to participation on the EAP were specified for systemic and loco-regional therapy.^12^

Patients did not routinely receive any prophylactic pre-medication with corticosteroids or other supportive medication but could do so in response to previous episodes of CRS.

All submitting centers satisfied local institutional arrangements for anonymized data sharing.

### Data collection, primary and secondary outcomes

Data was requested in a standardized, anonymized format from participating centers including demographic data on age, sex and ECOG PS; data on disease characteristics including site of metastases and burden of liver metastases (M1a, b or c); baseline biochemical parameters including LDH, alkaline phosphatase, and gamma glutyl-transferase (GGT); data on disease time course including time from first diagnosis of uveal melanoma to diagnosis of mUM, and time from diagnosis of metastatic disease to treatment with tebentafusp; data on prior systemic therapy (anti-PD(L)1 alone, anti-CTLA4 alone, none or combined) and loco-regional therapies (ablation, chemo-embolization, surgery, radiotherapy, chemoperfusion, none); data on best radiographic response during treatment; and data on toxicities from each dose of tebentafusp up to dose 10.

Best radiographic response during treatment was assessed locally and reported under the following categories: complete response (CR), partial response (PR), stable disease (SD), and progressive disease (PD). RECIST 1.1 reporting was not required. CRS was graded according to the American Society for Transplantation and Cell Therapy consensus grading,^13^ with rash graded as per version 5 of the Common Terminology Criteria for Adverse Events.

Our primary aim was to assess the efficacy of tebentafusp in a real-world setting, with a secondary aim of ascertaining toxicities. The main efficacy measures were mOS (time from start of tebentafusp treatment to death from any cause or last follow-up) and mPFS (time from start of tebentafusp treatment to locally determined radiological progression). A further efficacy measure was the best radiological response at any point during treatment (CR, PR, SD, or PD).

As a measure of the burden of toxicity and associated supportive care requirements, we collected data on the percentage of patients with grade 1-4 CRS and the percentage of patients requiring supportive corticosteroids or fluids for the first ten doses of tebentafusp per patient. Other measures of interest were the percentage of patients with a grade 3 or 4 rash after each dose and the duration of hospital stay for doses 1 to 3 during the dose escalation phase.

As data on some parameters were not available for all patients within the cohort, for any given feature of interest, the percentage shown is calculated as a percentage of the number for whom data on that feature are available (*n*).

### Statistical analysis

All statistical analyses were performed using R software (version 4.3.0). Descriptive statistics summarized baseline demographic and clinical characteristics, with continuous variables presented as means and standard deviations or medians and interquartile ranges, depending on data distribution, and categorical variables summarized using counts and percentages.

Survival outcomes were assessed using Kaplan–Meier survival analysis via the survfit() function from the survival package (version 3.8-3), which computes 95% confidence intervals, and these were visualized with ggsurvplot() from the survminer package (version 0.5.0). Cox proportional hazards regression was conducted with coxph() in the survival package (version 3.8-3) to estimate hazard ratios and their 95% confidence intervals.

Categorical associations were evaluated with chi-square tests, switching to Fisher’s exact test when any expected cell count fell below five. Continuous–continuous relationships were explored by Spearman’s rank correlation. To limit false positives, all *P*-values for multiple comparisons were adjusted using the Benjamini–Hochberg procedure, and variables with an adjusted *P* < .05 and acceptable collinearity were selected for inclusion in the final multivariable Cox models. Whilst the lower bound of the 95% confidence interval could be calculated for our survival data, the upper bound could often not be calculated due to the low number of events once the median had been reached, and this is denoted as NR (not reached) in our data.

## Results

### The patient cohort and disease characteristics

We received data on 88 patients treated across 9 centers in the UK tebentafusp EAP, representing 77% of patients treated within the UK EAP. Full cohort characteristics are shown in Table 1. Male patients accounted for 56% of the cohort, with a cohort median age of 64 years (range 18-82 years). The median time from onset of metastatic disease to tebentafusp treatment was 1 year in our cohort (range 0-14 years). Most patients had a good functional baseline with a PS0 (60%) or PS1 (37%). Two patients on the EAP began treatment with a PS2, having declined functionally between consent and starting on therapy.

**Table 1.** Baseline characteristics of the UK tebentafusp EAP cohort.

Characteristics	*n* (%)
**Median age, (IQR), (range)**	64, (56-73), (18-82)
**Sex**	
** Female**	39 (44)
** Male**	49 (56)
**ECOG performance status**	
** 0**	49 (60)
** 1**	30 (37)
** 2**	2 (2)
**Disease timeline**	
** Time from primary diagnosis to start of treatment**	4 yrs (range 1-28)
** Time from primary diagnosis to metastatic disease**	3 yrs (range 0-18)
** Time from metastatic disease to start of treatment**	1 yr (range 0-14)
**LDH**	
** Normal**	34 (48)
** 1-2× ULN**	23 (32)
** ≥2× ULN**	14 (20)
**GGT**	
** Normal**	20 (54)
** 1-2× ULN**	10 (27)
** ≥2× ULN**	7 (19)
**Hepatic disease burden**	
** M1a (≤3 cm)**	42 (52)
** M1b (3.1-8 cm)**	16 (20)
** M1c (≥8.1 cm)**	23 (28)
**ALP**	
** Normal**	66 (75)
** 1-2× ULN**	14 (16)
** ≥2× ULN**	8 (9)
**Metastasis location**	
** Hepatic + Extra hepatic**	43 (49)
** Hepatic only**	40 (45)
** Extra hepatic only**	5 (6)
**Loco-regional treatment**	
** None**	58 (72)
** Ablation**	10 (12)
** Perfusion**	1 (1)
** Chemoembolization**	2 (2)
** Radiotherapy**	1 (1)
** Surgery**	8 (10)
**Systemic treatment**	
** None**	53 (71)
** Anti-CTLA4 and anti-PD(L)1**	15 (20)
** Anti-PD(L)1 monotherapy**	6 (8)
** Anti-CTLA4 monotherapy**	1 (1)

Baseline demographic, disease characteristics, and treatment data for patients treated on the UK tebentafusp EAP. For each categorical feature of interest, the number and percentage of patients known to be in each category are shown. As data on some features were not available for all patients within the cohort, for any given feature, the percentage shown is calculated as a percentage of the number for whom data on that feature are available. For age and time metrics, the median for the cohort is shown with range in brackets; additionally, the interquartile range is shown for cohort age.

Abbreviations: ALP, alkaline phosphatase; ECOS, Eastern Cooperative Oncology Group; GGT, gamma glutyl-transferase; LDH, lactate dehydrogenase; ULN, upper limit of normal.

94% had liver metastases, often as the solitary site of macroscopic metastatic disease (45%). The size of the largest liver lesion was used as a surrogate for burden of liver disease, ranging from M1a (largest lesion ≤3 cm) to M1c (largest lesion ≥8 cm). 52% of our patients had M1a disease, with 20% having M1b disease and 28% having M1c disease.

LDH, commonly used as a surrogate marker of overall disease burden, was normal in 48%, 1-2× ULN in 32%, and ≥2× ULN in 28% of patients.

Tebentafusp was first-line systemic therapy for 71% of our cohort (*n* = 53), with 20% (*n* = 15) of patients receiving prior doublet checkpoint-inhibitor immunotherapy with an anti-CTLA4 and anti-PD(L)1 agent, and fewer than 10% of patients having received single agent immunotherapy. A majority of patients (72% of patients, *n* = 58) were loco-regional treatment naïve, with ablation (12% of patients, *n* = 10) and surgery (10% of patients, *n* = 8) the most frequently used prior loco-regional therapies.

### Real-world treatment efficacy and factors impacting efficacy

**Figure 1.**
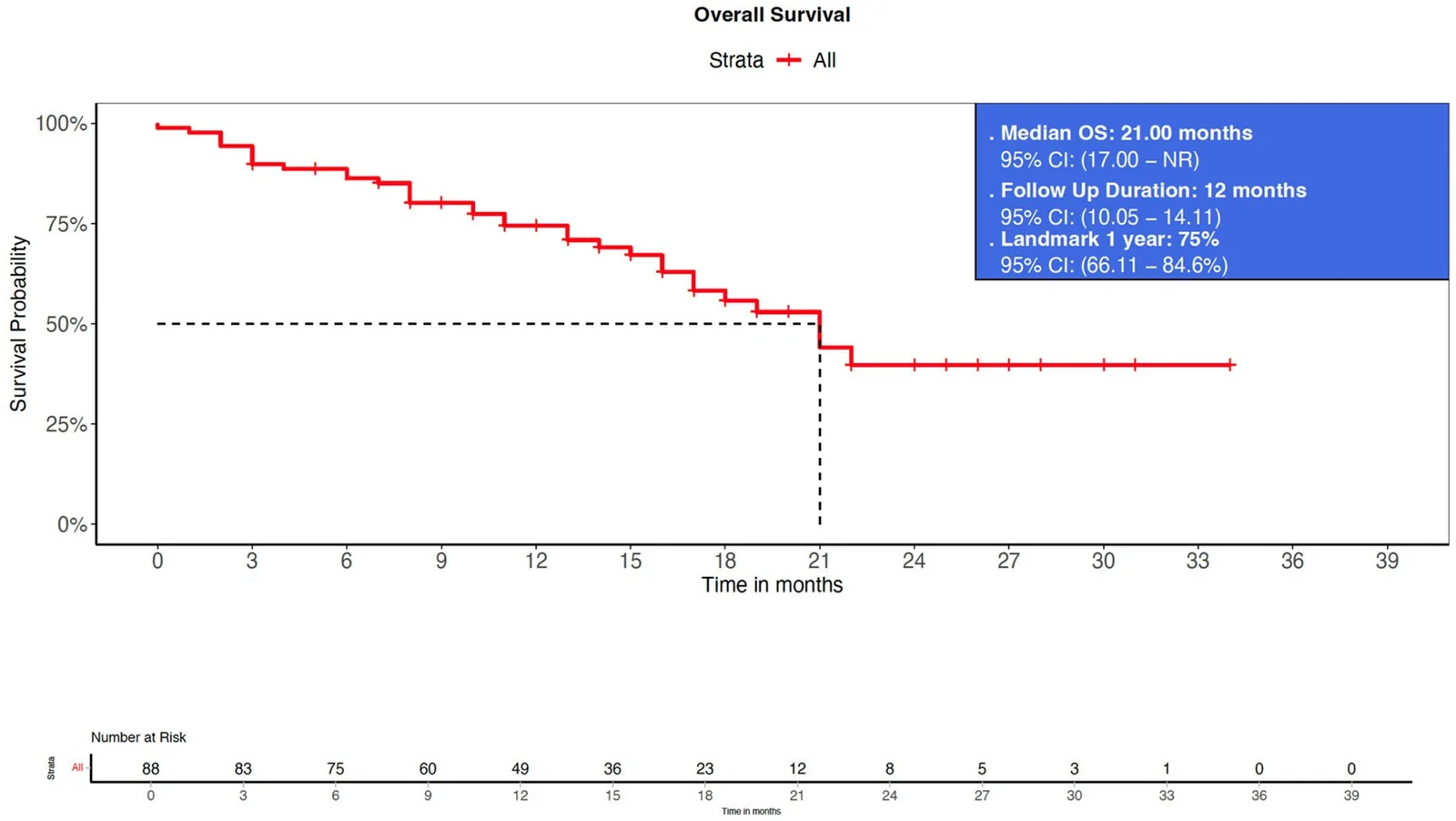
Overall survival for the UK tebentafusp EAP cohort. Overall survival for the cohort is shown as a Kaplan–Meier survival curve (*y* = % of cohort known to be alive at a specific time point, *x* = follow-up time in months). Bar below the main graph showing the number at risk at each point in time. The dashed lines show the median overall survival (OS) intersection point on the curve. The inset box describes key metrics, including cohort median OS, median duration of follow-up in the cohort, and the 1-year survival rate with the 95% CI of each shown below the main metric.

**Figure 2.**
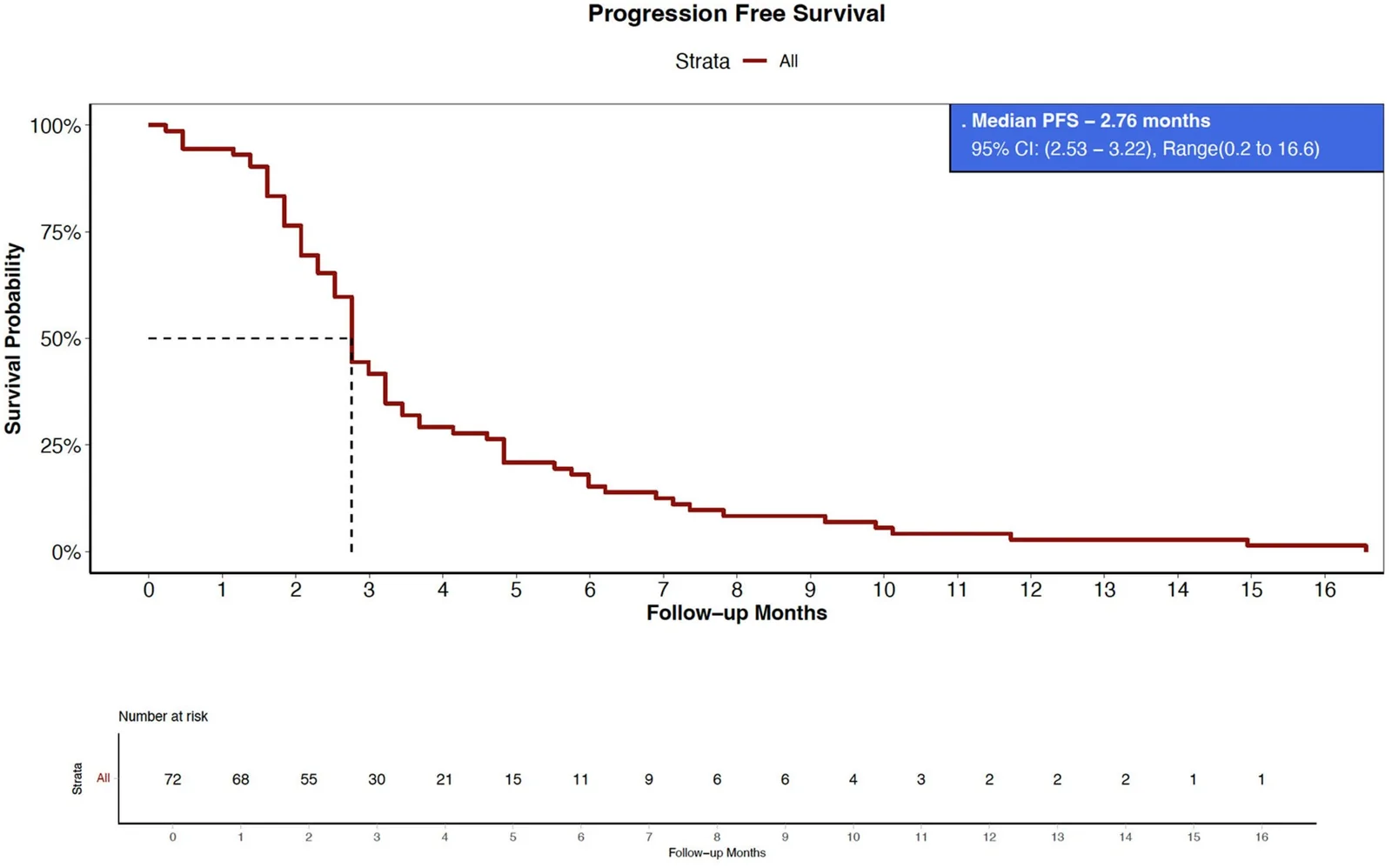
Progression-free survival for the UK tebentafusp EAP cohort. Progression-free survival for the cohort is shown as a Kaplan–Meier survival curve (*y* = % of cohort known to be progression-free at a specific time point, *x* = follow-up time in months). Bar below the main graph showing the number at risk at each point in time. The dashed lines show the median progression-free survival (PFS) intersection point on the curve. The inset box shows the cohort median PFS, the 95% CI for median PFS, and the PFS range in the cohort.

Median follow-up duration of patients was 12 months (range 0-34 months), with a survival rate of 75% (95% CI, 66.11-84.6) at the 12-month time point (Figure 1). Despite ∼30% of our patients being treated in the second-line, the mOS of our cohort closely mirrored data from the first-line IMCgp100-202 trial at 21 months (95% CI, 17 months to NR, Figure 1), with a mPFS of 2.76 months (95% CI, 2.53-3.22 months; Figure 2).

15% of our patient cohort had radiological regression of disease (PR 13%, CR 2%; Figure S1). Around half of our cohort (49%) had SD as their best response, and 35% of patients had PD as their best response (Figure S1). Of note, in those with PD as their best radiological response, we observed an mOS of 14 months (95% CI, 11 months to NR; Figure S2), comparable to first-line trial data.

We also examined factors expected to impact treatment outcomes and compared overall survival in our cohort with patients split by sex, median age (≤64 or >64), or the sites of metastatic disease (extra-hepatic only, hepatic only, or intra-and extra-hepatic; Figures S3-S5). No clear differences in survival were observed.

**Figure 3.**
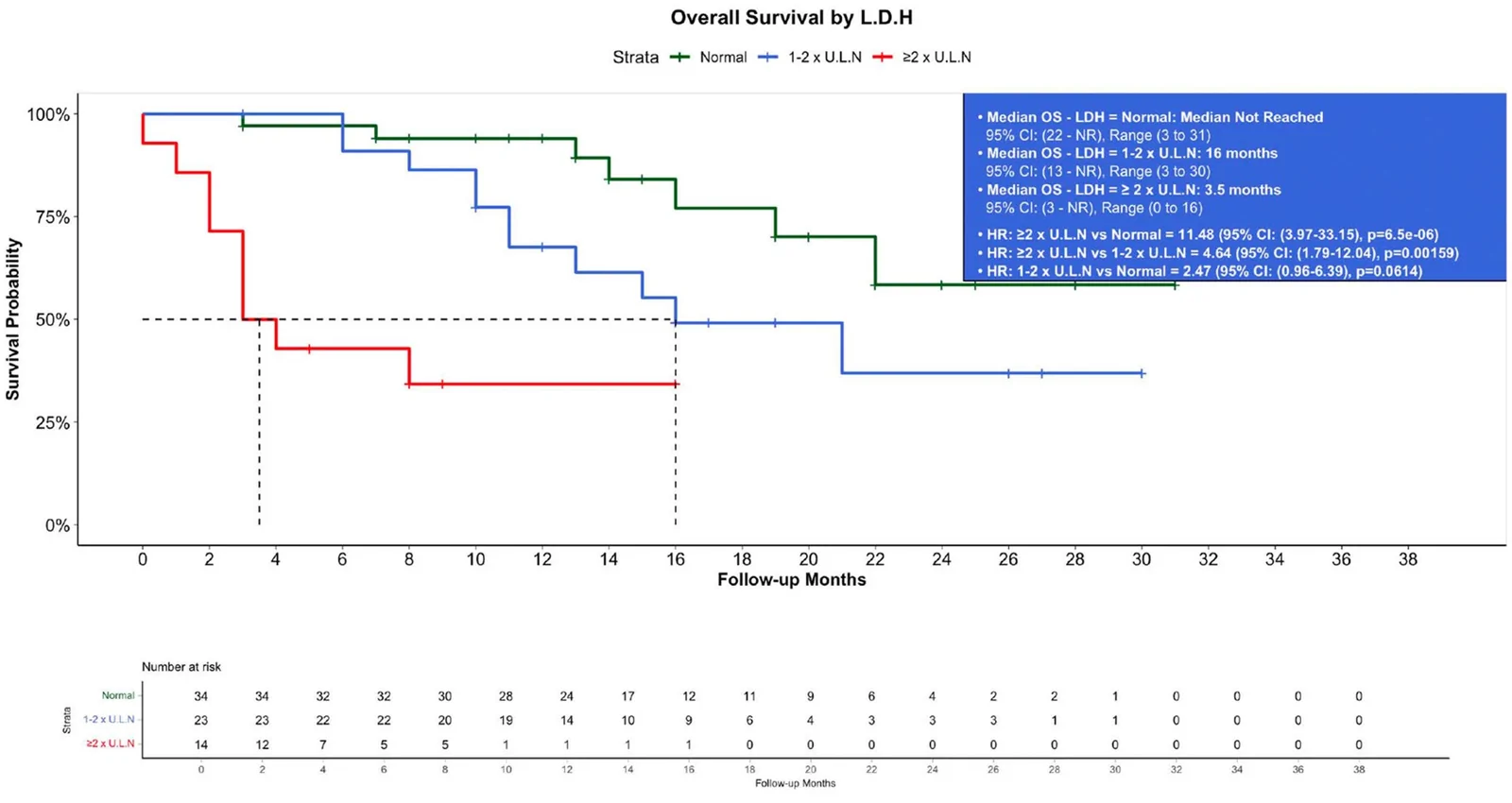
Overall survival in relation to disease burden as measured by LDH. Overall survival for patients split by baseline LDH levels—LDH normal (green curve), LDH 1-2× ULN (blue curve), and LDH ≥ 2× ULN (red curve)—shown as separate Kaplan–Meier survival curves on the same graph (*y* = % of patients in each subset known to be alive at a specific time point, *x* = follow-up time in months). Bar below the main graph showing the number at risk at each point in time for each subset. The dashed lines show the median overall survival (OS) intersection point on the curve for each subset where this is reached. The inset box describes the median OS, the 95% CI for the median OS, and the OS range for each subset of patients. Also shown in the inset box is the hazard ratio for death for pairwise comparisons of the subsets, showing a significantly higher risk of death for those with a baseline LDH ≥ 2× ULN when compared to those with a normal LDH or LDH 1-2× ULN at baseline.

LDH levels showed a marked inverse relationship with survival. The mOS was not reached in those with a normal LDH (95% CI, 22 months to NR), compared to 16 months (95% CI, 13 months to NR) in those with an LDH 1-2× ULN, and 3.5 months (95% CI, 3 months to NR) in those with an LDH ≥ 2× ULN (Figure 3). A significant difference in the HR for risk of death was observed for those with an LDH ≥ 2× ULN, compared to those with a normal LDH (HR 11.48, 95% CI, 3.97-33.15, *P* = 6.5 × 10^−6^) and an LDH 1-2× ULN (HR 4.64, 95% CI, 1.79-12.04, *P* = .00159) (Figure 3).

**Figure 4.**
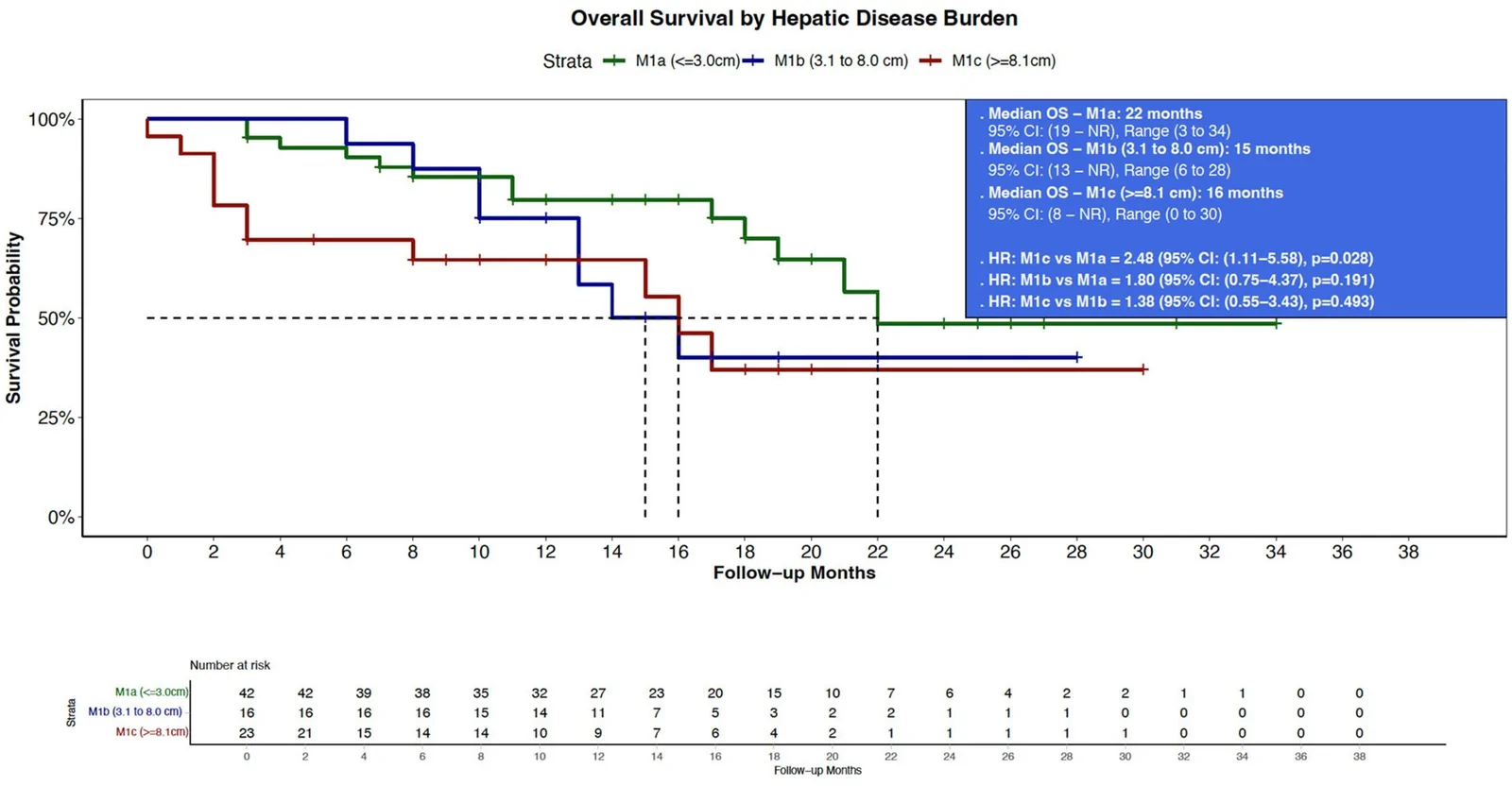
Overall survival in relation to liver metastatic disease burden. Overall survival for patients split by liver metastatic burden at baseline—M1a (green curve), M1b (blue curve), and M1c (red curve)—shown as separate Kaplan–Meier survival curves on the same graph (*y* = % of patients in each subset known to be alive at a specific time point, *x* = follow-up time in months). Bar below the main graph showing the number at risk at each point in time for each subset. The dashed lines show the median overall survival (OS) intersection point on the curve for each subset. The inset box describes the median OS, the 95% CI for the median OS, and the OS range for each subset of patients. Also shown in the inset box is the hazard ratio for death for pairwise comparisons of the subsets, showing a significantly higher risk of death for those with the highest burden of disease (M1c) compared to those with the lowest burden of disease (M1a).

There was a significant positive correlation between LDH and liver M-stage (data not shown). A trend toward longer survival in those with a lower burden of liver metastatic disease was also noted, with patients with M1a disease having a markedly longer mOS (22 months, 95% CI, 19 months to NR), compared to those with M1b (mOS 15 months, 95% CI, 13 months to NR) or M1c disease (mOS 16 months, 95% CI, 8 months to NR; Figure 4). Those with the highest burden M1c disease had a significantly higher risk of death (HR for death 2.48, 95% CI, 1.11-5.58, *P* = .028), compared to those with the lowest burden M1a disease.

In addition to burden of disease, performance status showed clear impacts on treatment outcomes with the mOS not reached (95% CI, 18 months to NR) in the patient cohort with a PS0, compared to a mOS of 17 months (95% CI, 11 months to NR) in those with a PS1 (Figure S6). Those with a performance status of 1 had a significantly higher risk of death (HR 2.25, 95% CI, 1.11-4.60, *P* = .025) compared to those with a PS0 (Figure S6). The 2 patients with PS2 were excluded from this analysis due to the very small size of this group.

Given current interest in the sequencing of tebentafusp, we compared outcomes for patients treated in the first-line with patients treated in the second-line and beyond. First-line tebentafusp was associated with a longer mOS of 22 months (95% CI, 18 months to NR), compared to a mOS of 16 months (95% CI 10 months to NR) in the second-line and beyond (Figure S7). When comparing second-line with first-line treatment, the HR for death was significantly in favor of first-line treatment (HR 2.09; 95% CI, 1-4.35, *P* = .0488; Figure S7). However, mPFS was identical at 2.8 months for both first- and second-line cohorts, with a non-significant HR for progression (HR 1.01; 95% CI, 0.57-1.77, *P* = .981; Figure S8).

Patients who had received prior loco-regional therapy had a numerically longer mOS (mOS not reached: 95% CI, NR-NR) than those receiving tebentafusp without prior loco-regional treatment (mOS 19 months: 95% CI, 15 months to NR; Figure S9), although the HR for death which favors prior loco-regional therapy was not significant (HR 0.34; 95% CI, 0.10-1.14, *P* = .080; Figure S9).

### Treatment-related toxicity, associated factors, and management of toxicities

Due to the potential for CRS, doses 1 to 3 during the dose escalation phase are delivered during an inpatient stay with overnight observation as per the summary of product characteristics. The requirement for inpatient care and duration of hospital stay generally reduced with successive doses. In our cohort, 43.7% of patients required only one inpatient night stay post dose 1, increasing to 59.5% for dose 2 and 66.7% for dose 3. Those requiring 3 or more nights as an inpatient similarly fell from 20.7% for dose 1 to 10.7% for dose 2 and 7.4% for dose 3 (Table S1). The median cumulative number of nights stayed was 3 nights over doses 1 to 3 (range 0-11).

**Figure 5.**
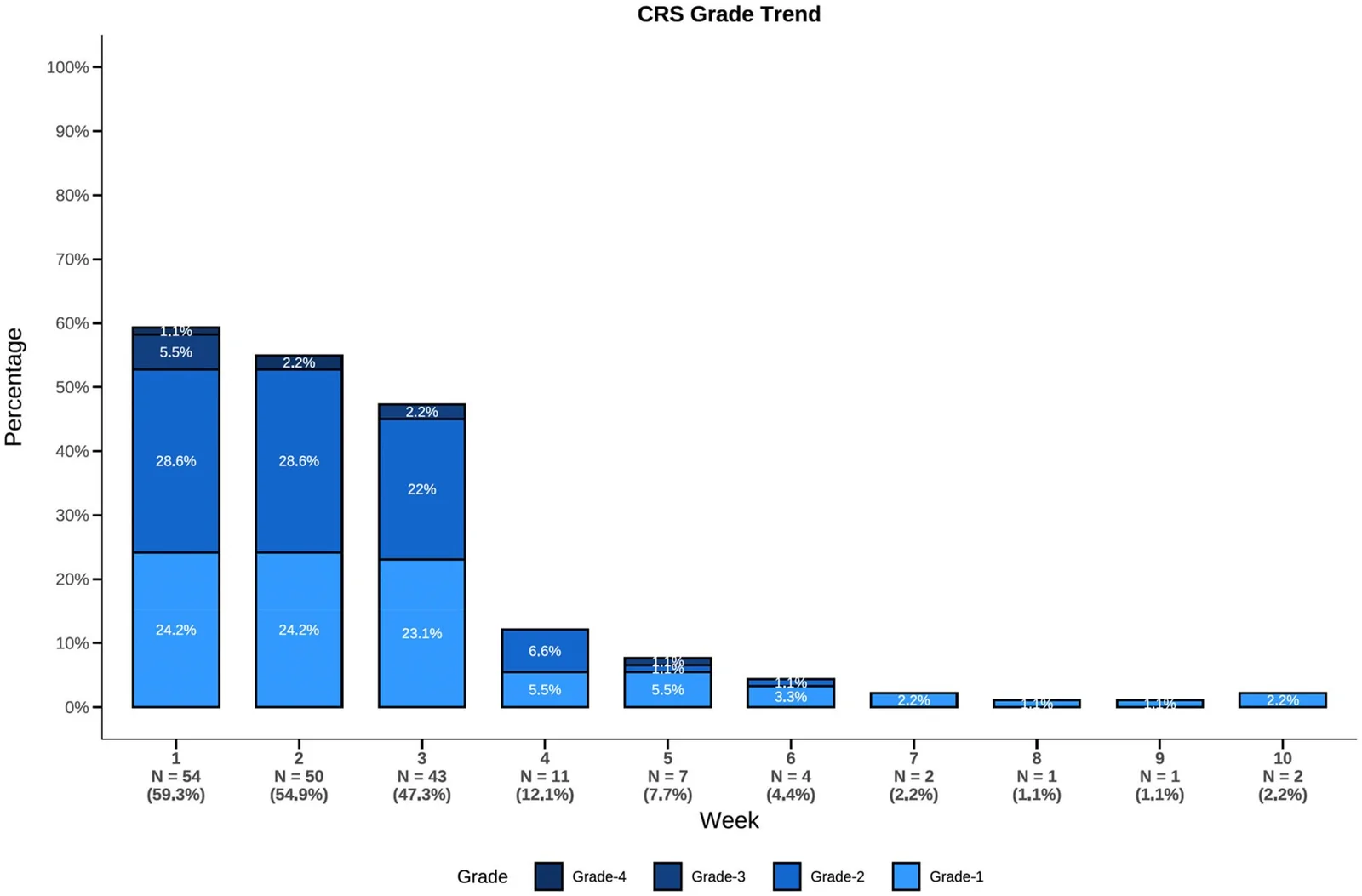
All grade CRS are less frequent with consecutive doses of tebentafusp. Stacked bar chart showing decreasing incidence of CRS with each successive dose for doses 1 to 10 of tebentafusp. Each bar represents a single dosing time point, with dose 1 to dose 10 shown along the *x*-axis. The height of the bar (*y*-axis) represents the cumulative percentage of patients experiencing CRS, with bar segments showing the percentage of patients with each grade of CRS per dose. Colors denoting grade as shown in the color key below the graph.

Both all grade CRS and grade 3/4 CRS reduced in frequency with each successive dose (Figure 5). Grade 3 or 4 CRS occurred in 6.6% of patients post-dose 1 and 2.2% of patients post doses 2 and 3, with no grade 3 or 4 CRS beyond dose 5 in any patient (Figure 5). Similarly, 38.6% of patients required intravenous fluids and 22.7% of patients required corticosteroids post dose 1, whilst fewer than 5% of patients required either intravenous fluids or corticosteroids beyond dose 4 (Table S2). No patients within our cohort required tocilizumab for CRS.

**Figure 6.**
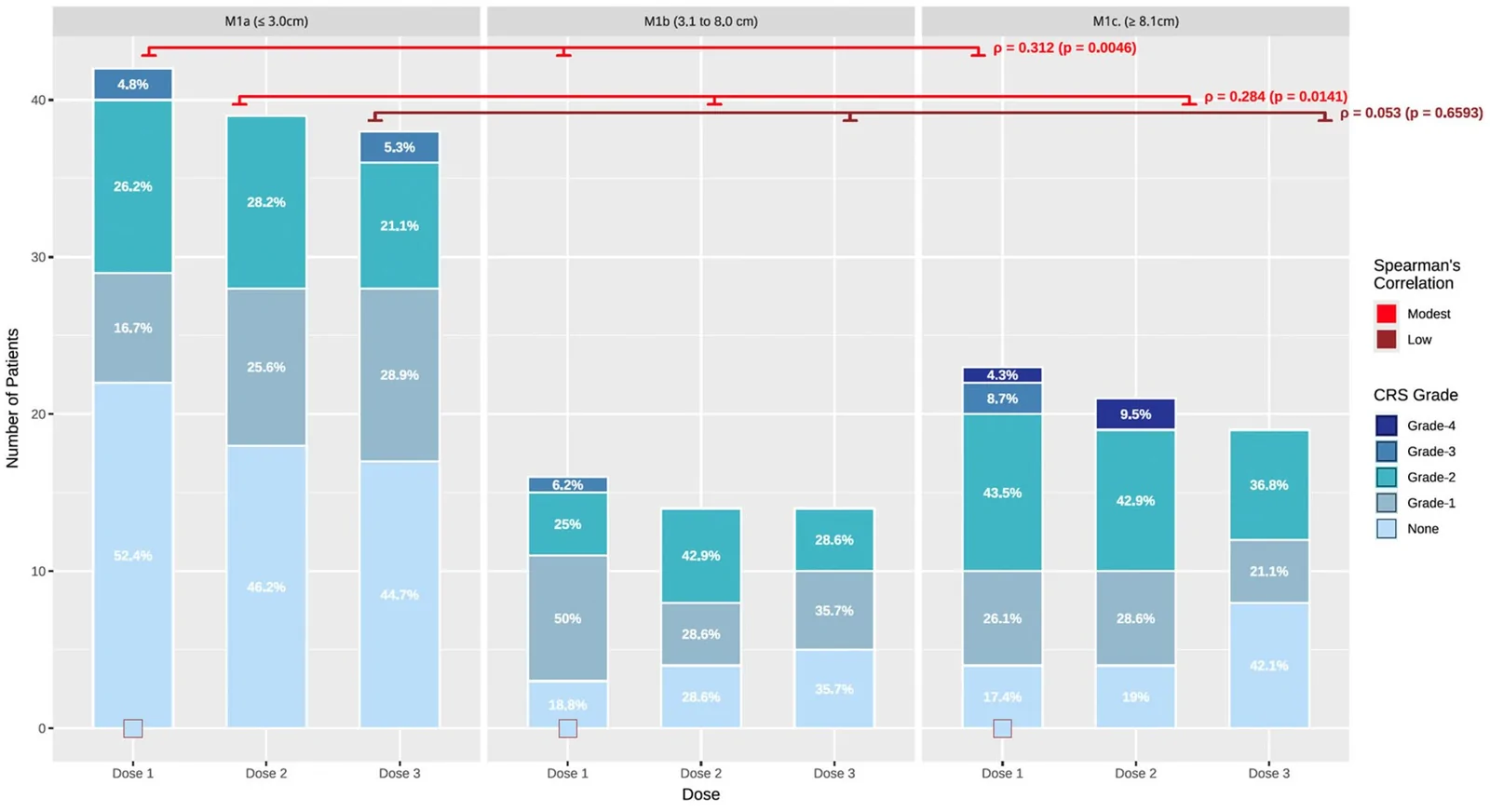
Higher liver metastatic burden carries greater risk of CRS. Stacked bar chart with patient cohort split by M-stage into three panels (from left to right: M1a, M1b, and M1c). Within each panel, each bar represents a single dosing time point, with dose 1 to dose 3 shown along the *x*-axis. The height of the bar (*y*-axis) represents the total number of patients for that M-stage and dosing time point. The bar segments and labels show the percentage of patients with each grade of CRS for that M-stage and dosing time point, with segments for no CRS (grade 0) also shown. Colors denoting grade as shown in the color key to the right of the graph. Values adjacent to red significance bars show Spearman correlation between M-stage and incidence of CRS, with adjusted *P*-values in brackets, for each of doses 1 to 3. There is a modest but significant positive correlation between M-stage and incidence of CRS for doses 1 and 2.

The burden of metastatic disease in the liver (M1a-c) was the only factor found to correlate significantly with the occurrence of CRS. Increasing liver metastatic burden showed a modest but significant positive correlation with the incidence of CRS for dose 1 (Spearman *r* = 0.312, *P* = .0046) and dose 2 (Spearman *r* = 0.284, *P* = .0141) (Figure 6). This correlation remained significant after *P*-value adjustment for multiple testing (Figure 6), suggesting that those with higher burdens of liver metastatic disease experience both less efficacy from treatment (Figures 3 and 4) and greater risk of CRS (Figure 6).

Considering delayed toxicities, a grade 3 or 4 rash was seen in 13.6%-14.8% of patients post doses 1 to 3, but only in 0%-3.4% of patients from dose 4 and beyond (Table S2). Given the low frequency of grade 3/4 rash, factors predisposing to this could not be reliably examined.

## Conclusion

Tebentafusp is the first systemic therapy with a proven OS benefit in mUM.^7^ Here, we present data from 88 patients treated on the UK EAP for tebentafusp in mUM, with a focus on real-world efficacy and toxicity of tebentafusp in this disease setting. Our patient numbers and treatment outcomes are comparable to published retrospective studies from France (*n* = 72),^10^ Germany/Switzerland (*n* = 78)^9^ and the multicenter, international EURACAN study (175 patients across 4 centers).^11^

Baseline demographics in our predominantly treatment-naive cohort (Table 1) were comparable to the tebentafusp arm of the first-line IMCgp100-202 trial, with a similar sex distribution (55% male in the EAP cohort vs 51% in the trial) and comparable median age (64 years in both the EAP cohort and the trial).^7^ As might be expected, our real-world cohort comprised a greater proportion of patients with reduced performance status (37% PS1 in the EAP cohort vs 19% in the tebentafusp arm of IMCgp100-202 trial).

Liver metastases were near ubiquitous in both our EAP cohort (94% of patients) and the tebentafusp arm of the IMCgp100-202 trial (95% of patients), with similar proportions of patients with M1a disease (54% vs 55% respectively). However, patients with M1c disease and the greatest burden of liver metastases were over-represented in our EAP cohort (27% in EAP cohort vs 8% of patients in tebentafusp arm of IMCgp100-202 trial).

The main prognostic factor for overall survival in our cohort was LDH, with a baseline LDH ≥2× ULN carrying a significantly greater risk of death than a normal baseline LDH. A similar relationship was identified in a recent mUM meta-analysis and the real-world, retrospective EURACAN study.^2^^,^^11^ In our cohort LDH was significantly correlated with liver metastatic burden, as measured by the size of the largest lesion. In keeping with this, lower liver metastatic burden showed a trend toward improved survival.

The highest mOS of 22 months was observed in those with low burden M1a disease, mirroring data from the first-line IMCgp100-202 trial where the M1a cohort showed the greatest benefit from tebentafusp, though the HR favored tebentafusp treatment in all M-stage cohorts.^7^ Of note, a high burden of liver metastatic disease was also a significant predictor of risk of CRS during the dose escalation phase, suggesting the utility of this factor in assessing both risk and benefit from treatment.

Perhaps reflective of the functional consequences of a higher burden of disease, performance status showed clear impacts on treatment outcomes, with those having a PS0 at the start of treatment having a longer mOS than those who had a PS1 at the start of treatment. The greater efficacy seen in those with a low burden of disease and good performance status also provides support for using tebentafusp earlier in the metastatic disease setting.

The mOS and mPFS measures in our overall EAP cohort are very comparable to both first-line trial data^7^ and real-world data from patients treated with tebentafusp across Europe,^9^^,^^11^ suggesting our outcomes from the UK EAP reflect broad real-world efficacy of tebentafusp. This is striking given that ∼30% of patients in both our UK EAP and other real-world cohorts were treated in the second line.

The resource utilization associated with tebentafusp treatment during the dose escalation phase is a significant consideration for centers who are considering offering this therapy. The manageable toxicity profile of tebentafusp and reducing incidence of toxicity with cumulative doses, are well characterized in the trial setting.^14^ Our study reports the incidence of the most common toxicities of CRS and rash for each of the doses 1 to 10 and provides the first real-world confirmation of diminishing longitudinal toxicities. Additionally, the need for supportive medication and duration of inpatient stays for each dose are described in detail to guide resource planning.

The required inpatient stay within the dose escalation phase was generally brief, with a median of 3 cumulative number of nights as an inpatient for doses 1 to 3. Admission precautions for doses 1 to 3 appropriately mitigated the greatest period of risk for patients, with treatment beyond dose 5 associated with minimal toxicities. Significantly, no routine premedication was used in this cohort. The use of corticosteroids to manage CRS did not appear to have any impact on mOS, which was very comparable to first-line trial data. Furthermore, analysis of the impact of corticosteroids on outcomes from the phase III IMCgp100-202 trial suggests no adverse effect on survival from use of corticosteroids.^15^

Our analysis of patients treated in the second line with tebentafusp on the EAP showed, as expected, a lower mOS of 16 months. This mOS closely mirrors second-line trial data, with the IMCgp100-102 trial demonstrating an mOS of 16.8 months in tebentafusp treated patients.^6^

Whilst some groups have reported a longer real-world mPFS and mOS for patients treated with tebentafusp in the second-line setting,^6^^,^^9^ these studies are limited by the small patient numbers and marked differences between patient cohorts treated in the first and second lines. These include differences in distribution of disease^9^ and number of prior lines of treatment.^16^ Of note, in the EURACAN study which is the largest real-world dataset in this setting, no significant differences were found in mOS or mPFS between those treated in the first and second lines.^11^ By contrast, the mPFS and mOS for patients treated with first-line tebentafusp in the UK EAP exceeded that for those treated in the second-line setting, reflecting the first- and second-line trial data.^6–8^ The hazard ratio for risk of death was also significant, favoring first-line tebentafusp, though the confounding factors in our small, highly heterogeneous patient population mean it has to be interpreted with caution.

Prior meta-analyses have suggested significant surveillance and selection bias in the trial outcomes seen post loco-regional and systemic therapies in mUM.^1^^,^^2^ It is also possible that the higher mOS for tebentafusp treatment seen in the second-line setting in some real-world datasets reflects selection bias for a more indolent disease biology in the second-line setting, with a previous Dutch real-world analysis finding significant selection bias within routine clinical care, outside of trial settings.^17^ Patients who have relatively stable or oligometastatic disease may be over-represented in second-line trials, being well enough for second-line treatment on progression, with patients who have an adverse disease biology being lost due to lack of fitness. This would additionally explain the finding that patients who had received prior loco-regional therapy appear to be a favorable prognosis cohort.

In summary, the UK EAP showed a remarkably similar mOS and mPFS when compared to the first-line line IMCgp100-202 trial of tebentafusp.^7^^,^^8^ Importantly, treatment was well tolerated with predictable and manageable toxicities. This real-world dataset describing the efficacy and toxicity of this exciting new treatment will hopefully inform centers considering offering this treatment to their population and aid in their resource planning for the same.

## Supplementary Material

oyag341_Supplementary_Data

## Data Availability

The raw data supporting the findings in this article will be shared on reasonable request to the corresponding author.
