# Supplementary material for: Real-world efficacy and toxicity of tebentafusp in metastatic uveal melanoma patients treated on the United Kingdom expanded access programme
Source: Oncologist. 2026 Aug 25;31(10):oyag341. doi: 10.1093/oncolo/oyag341 (PMC13568816; doi:10.1093/oncolo/oyag341)
Supplement: Supplementary file 1 [file oyag341_supplementary_data.zip › Revised UK Tebentafusp EAP Supplementary Information.docx]

**Supplementary Figure 1: Best radiological response in the UK EAP cohort**

Doughnut chart showing the best radiological outcomes for patients in the cohort with each segment representing proportion of the cohort in each of the following categories: stable disease (S.D), progressive disease (P.D), partial response (P.R) and complete response (C.R). The percentage of patients in each subset is stated on the relevant segment of the doughnut chart.

**Supplementary Figure 2: Overall survival in patients with progressive disease as best radiological response**

Overall survival for patients with progressive disease as best radiological response, shown as a Kaplan-Meier survival curve (y = % of cohort known to be alive at a specific time point, x = follow up time in months). Bar below the main graph showing the number at risk at each point in time. The dashed lines show the median overall survival (OS) intersection point on the curve. The inset box describes median OS, the 95% CI for median OS and OS range in this subset of patients.

**Supplementary Figure 3: Overall survival by sex**

Overall survival for male (red curve) and female (blue curve) patients, shown as separate Kaplan-Meier survival curves on the same graph (y = % of male/female patients known to be alive at a specific time point, x = follow up time in months). Bar below the main graph showing the number at risk at each point in time for both subsets. The dashed lines show the median overall survival (OS) intersection point on the curve for male and female subsets. The inset box describes median OS, the 95% CI for median OS and OS range for each subset of patients. Also shown in the inset box are the 1-year survival rates for each subset and the 95% CI for this value; as well as the hazard ratio for death for males compared to females, with no significant difference observed.

**Supplementary Figure 4: Overall survival by age cohorts split on median age**

Overall survival for patients > 64 years (yellow curve) and patients ≤ 64 years (green curve), shown as separate Kaplan-Meier survival curves on the same graph (y = % of patients >64/≤64 years known to be alive at a specific time point, x = follow up time in months). Bar below the main graph showing the number at risk at each point in time for both subsets. The dashed lines show the median overall survival (OS) intersection point on the curve for both subsets. The inset box describes median OS, the 95% CI for median OS and OS range for each subset of patients. Also shown in the inset box is the hazard ratio for death for those >64 compared to those ≤64 years, with no significant difference observed.

**Supplementary Figure 5: Overall survival by metastatic disease sites**

Overall survival for patients with extra-hepatic disease only (blue curve), patients with hepatic- and extra-hepatic disease (red curve) and patients with hepatic disease only (green curve), shown as separate Kaplan-Meier survival curves on the same graph (y = % of patients in each subset known to be alive at a specific time point, x = follow up time in months). Bar below the main graph showing the number at risk at each point in time for each subset. The dashed lines show the median overall survival (OS) intersection point on the curve for each subset. The inset box describes median OS, the 95% CI for median OS and OS range for each subset of patients. Also shown in the inset box is the hazard ratio for death comparing the two most numerous subsets- those with intra- and extra-hepatic disease compared to those with hepatic disease only, with no significant difference observed.

**Supplementary Figure 6: Overall survival by performance status**

Overall survival for patients with a PS0 (yellow curve) and patients with PS1 (blue curve), shown as separate Kaplan-Meier survival curves on the same graph (y = % of patients PS0/PS1 years known to be alive at a specific time point, x = follow up time in months). Bar below the main graph showing the number at risk at each point in time for both subsets. The dashed lines show the median overall survival (OS) intersection point on the curve for each subset where this is reached. The inset box describes median OS, the 95% CI for median OS and OS range for each subset of patients. Also shown in the inset box is the hazard ratio for death for those with a PS1 compared to those of PS0, with a significantly greater overall survival observed for those with PS0.

**Supplementary Figure 7: Overall survival by tebentafusp sequencing**

Overall survival for patients receiving tebentafusp first-line (red curve) and patients receiving tebentafusp second-line (green curve), shown as separate Kaplan-Meier survival curves on the same graph (y = % of patients receiving first-line/second-line tebentafusp known to be alive at a specific time point, x = follow up time in months). Bar below the main graph showing the number at risk at each point in time for both subsets. The dashed lines show the median overall survival (OS) intersection point on the curve for each subset. The inset box describes median OS, the 95% CI for median OS and OS range for each subset of patients. Also shown in the inset box is the hazard ratio for death for those treated in the second-line compared to those treated with first-line tebentafusp, with a significantly greater survival observed for those treated in the first-line setting.

**Supplementary Figure 8: Progression-free survival by tebentafusp sequencing**

Progression-free survival for patients receiving tebentafusp first-line (green curve) and patients receiving tebentafusp second-line (yellow curve), shown as separate Kaplan-Meier survival curves on the same graph (y = % of patients receiving first-line/second-line tebentafusp known to be progression-free at a specific time point, x = follow up time in months). Bar below the main graph showing the number at risk at each point in time for both subsets. The dashed lines show the median progression-free survival (PFS) intersection point on the curve for each subset. The inset box describes median PFS and the 95% CI for median PFS for each subset of patients. Also shown in the inset box is the hazard ratio for risk of progression for those treated in the second-line compared to those treated with first-line tebentafusp, with no significant difference seen.

**Supplementary Figure 9: Overall survival by loco-regional therapy prior to tebentafusp**

Overall survival for patients without prior loco-regional treatment (red curve) and patients with history of previous loco-regional treatment (green curve), shown as separate Kaplan-Meier survival curves on the same graph (y = % of patients with/without prior locogreional treatment known to be alive at a specific time point, x = follow up time in months). Bar below the main graph showing the number at risk at each point in time for both subsets. The dashed lines show the median overall survival (OS) intersection point on the curve for each subset. The inset box describes median OS, the 95% CI for median OS and OS range for each subset of patients. Also shown in the inset box is the hazard ratio for death for who have received prior loco-regional therapy compared to those with no prior loco-regional therapy. Though a numerically longer median OS is seen those receiving prior loco-regional therapy, no significant difference in risk of death is observed.

**Supplementary table 1: Number of inpatient nights during dose escalation phase**

| **Dose of Treatment** | **Median nights in hospital (range)** | **% of patients staying 1 night** | **% of patients**  **staying 2 nights** | **% of patients staying ≥3 nights** |
| --- | --- | --- | --- | --- |
| **Dose 1- 20mcg (n=87)** | **2 (1-8)** | **43.7%** | **35.6%** | **20.7%** |
| **Dose 2- 30mcg (n=84)** | **1 (1-5)** | **59.5%** | **29.8%** | **10.7%** |
| **Dose 3- 68mcg (n=81)** | **1 (1-4)** | **66.7%** | **25.9%** | **7.4%** |
| **Doses 1-3 Cumulative** | **3 (1-11)** | **-** | **-** | **-** |

Table showing inpatient admission duration metrics for doses 1-3. Column 1 shows the dose and number of patients with data available on inpatient stay per dose. Column 2 shows median duration of inpatient stay per dose (range in brackets), and a median cumulative duration of inpatient stay for doses 1-3 (range in brackets). Columns 3-5 show the percentage of patients staying for 1, 2 and ≥3 nights respectively after each dose.

| **Dose** | **IV fluid support (%)** | **Corticosteroid support (%)** | **Rash G3/G4 (%)** |
| --- | --- | --- | --- |
| **1** | **38.6** | **22.7** | **13.6** |
| **2** | **36.4** | **18.2** | **13.6** |
| **3** | **27.3** | **10.2** | **14.8** |
| **4** | **10.2** | **1.1** | **3.4** |
| **5** | **3.4** | **2.3** | **0** |
| **6** | **1.1** | **1.1** | **0** |
| **7** | **1.1** | **1.1** | **1.1** |
| **8** | **1.1** | **1.1** | **1.1** |
| **9** | **1.1** | **2.3** | **1.1** |
| **10** | **1.1** | **1.1** | **1.1** |

**Supplementary Table 2: Diminishing need for supportive measures and incidence of grade 3/4 rash with consecutive doses of tebentafusp**

Percentage of the patient cohort requiring IV fluid support, requiring corticosteroids or presenting with grade 3/4 rash after each dose of treatment for doses 1 to 10. Diminishing requirement for supportive medications and decreasing incidence of grade 3/4 rash seen with cumulative doses.
